# Computational homogenization of histological microstructures in human prostate tissue: Heterogeneity, anisotropy and tension‐compression asymmetry

**DOI:** 10.1002/cnm.3758

**Published:** 2023-07-21

**Authors:** Calum Anderson, Chara Ntala, Ali Ozel, Robert L. Reuben, Yuhang Chen

**Affiliations:** ^1^ Institute of Mechanical, Process and Energy Engineering, School of Engineering and Physical Sciences Heriot‐Watt University Edinburgh UK; ^2^ Department of Pathology, Western General Hospital University of Edinburgh Edinburgh UK

**Keywords:** histology, homogenization, multiscale modelling, prostate tissue, soft tissue mechanics

## Abstract

Human prostatic tissue exhibits complex mechanical behaviour due to its multiphasic, heterogeneous nature, with hierarchical microstructures involving epithelial compartments, acinar lumens and stromal tissue all interconnected in complex networks. This study aims to establish a computational homogenization framework for quantifying the mechanical behaviour of prostate tissue, considering its multiphasic heterogeneous microstructures and the mechanical characteristics of tissue constituents. Representative tissue microstructure models were reconstructed from high‐resolution histology images. Parametric studies on the mechanical properties of the tissue constituents, particularly the fibre‐reinforced hyper‐elasticity of the stromal tissue, were carried out to investigate their effects on the apparent tissue properties. These were then benchmarked against the experimental data reported in literature. Results showed significant anisotropy, both structural and mechanical, and tension‐compression asymmetry of the apparent behaviours of the prostatic tissue. Strong correlation with the key microstructural indices such as area fractions of tissue constituents and the tissue fabric tensor was also observed. The correlation between the stromal tissue orientation and the principal directions of the apparent properties suggested an essential role of stromal tissue in determining the directions of anisotropy and the compression‐tension asymmetry characteristics in normal human prostatic tissue. This work presented a homogenization and histology‐based computational approach to characterize the apparent mechanical behaviours of human prostatic or other similar glandular tissues, with the ultimate aim of assessing how pathological conditions (e.g., prostate cancer and benign prostatic hyperplasia) could affect the tissue mechanical properties in a future study.

## Graphical abstract

This study aims to establish a computational homogenization framework for quantifying the mechanical behaviour of prostate tissue, considering its multiphasic heterogeneous microstructures and the mechanical characteristics of tissue constituents. The results showed significant anisotropy, both structural and mechanical, and tension‐compression asymmetry of the apparent behaviours of the prostatic tissue. Strong correlation with the key microstructural indices such as area fractions of tissue constituents and the tissue fabric tensor was also observed. The correlation between the stromal tissue orientation and the principal directions of the apparent properties suggested an essential role of stromal tissue in determining the directions of anisotropy and the compression‐tension asymmetry characteristics in normal human prostatic tissue.

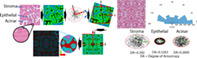

## 1 INTRODUCTION

Biological tissue often exhibits complex mechanical behaviours due to its multiphasic and heterogeneous nature, involving hierarchical microstructures across multiple length scales.^1^ In human prostate, this is further complicated by the mixture of structural and functional components which can be simplified as a muscular‐glandular tissue morphology.^2^ The gland structure consists of branches of acini, each including acinar lumens lined by secretory epithelial cells and surrounded by basal cells and basement membrane. The terminal acini are connected eventually with the urethra via a network of ducts, of overall shape rather like a ‘cauliflower’. These acinar structures are surrounded by fibromuscular stromal tissue, acting mechanically as a supporting matrix. For normal prostatic tissue, there are three major structural constituents that make a significant contribution to the overall mechanical properties; the stromal tissue, the epithelial compartments, consisting of basal and glandular epithelial cells, and the acinar lumen. Although not measured directly in prostate, there have been ample studies in the literature on the mechanical characterization of epithelial cells, most commonly involving the use of atomic force microscopy,^3^ tweezing and characterisation methods such as traction force microscopy.^4^ Similarly, stromal tissue and its role in tissue mechanical behaviours has also been extensively studied in breast and cornea.^5^, ^6^ Interestingly, stroma is known to present anisotropic characteristics, depending on the relative orientation of fibre groups and the applied load direction.^7^ The resulting structural and mechanical anisotropy in prostate tissue, combined with the asymmetric behaviours under tension and compression as a result of the presence of collagen fibres in stroma, have not been studied before.

To investigate how the underlying constituents affect the apparent mechanical behaviours of tissue, it is often necessary to establish the ‘structure–property relationship’ through explicit representation of tissue microstructure through a multiscale approach.^8^, ^9^ Attempts have been made to experimentally quantify the apparent mechanical properties, although mostly only elastic, of the prostatic tissue. Elastography, including ultrasound^10^, ^11^ and optical coherence^12^ elastography, has been used to estimate the elastic properties of prostate, whilst other studies^13^, ^14^, ^15^, ^16^ have used indentation tests on ex vivo prostates. Notwithstanding the different testing methods, characterization of the elastic properties of prostates is not a trivial task. This is because the significant level of tissue heterogeneity, intrinsic to the characteristics of prostate zonal anatomy,^17^ could give rise to variations in different regions of the prostate, and/or be dependent on the indenter size. Furthermore, the pathological conditions of the prostate, most commonly prostate cancer and benign prostate hyperplasia (BPH), are usually of clinical interest, and there could well be a significant difference between in vivo and ex vivo tissue responses. In our previous work,^18^ we studied how certain mixtures of normal and cancerous tissue could dictate the apparent tissue properties, although both tissue types were treated as homogeneous and isotropic thus lacking structural descriptions of tissue constituents and their detailed mechanical characteristics.

This study aims to establish a computational framework for quantifying the mechanical behaviour of human prostatic tissue, taking into account its multiphasic heterogeneous constituents and their complex mechanical characteristics. Representative tissue microstructural models, involving stroma, epithelial compartments and acinar lumens, will be reconstructed from high resolution histological images of excised prostate tissue. Specifically, parametric studies on the mechanical properties of tissue constituents will be carried out to quantify their effects on the apparent properties. In addition, both structural and mechanical anisotropy as well as the tension‐compression asymmetry arising from the stromal tissue are also investigated, to reveal the key microstructural indices that could dictate the structure–property relationship in prostatic tissue.

## 2 MATERIALS AND METHODS

### 2.1 Histology samples, image segmentation and model reconstruction

Hematoxylin and eosin (H&E) histology images for prostatic tissue were acquired from public research databases.^19^, ^20^ The selected images had a pixel resolution between of 0.25–0.49 μm. Multiple regions of normal prostatic tissue, confirmed by the uropathologist, were obtained using ImageScope software (Leica). Four regions of interest (ROIs) were randomly selected, from which a total number of 10 representative volume elements (RVEs) models were chosen. The optimization of RVE size will be discussed later.

**FIGURE 1:**
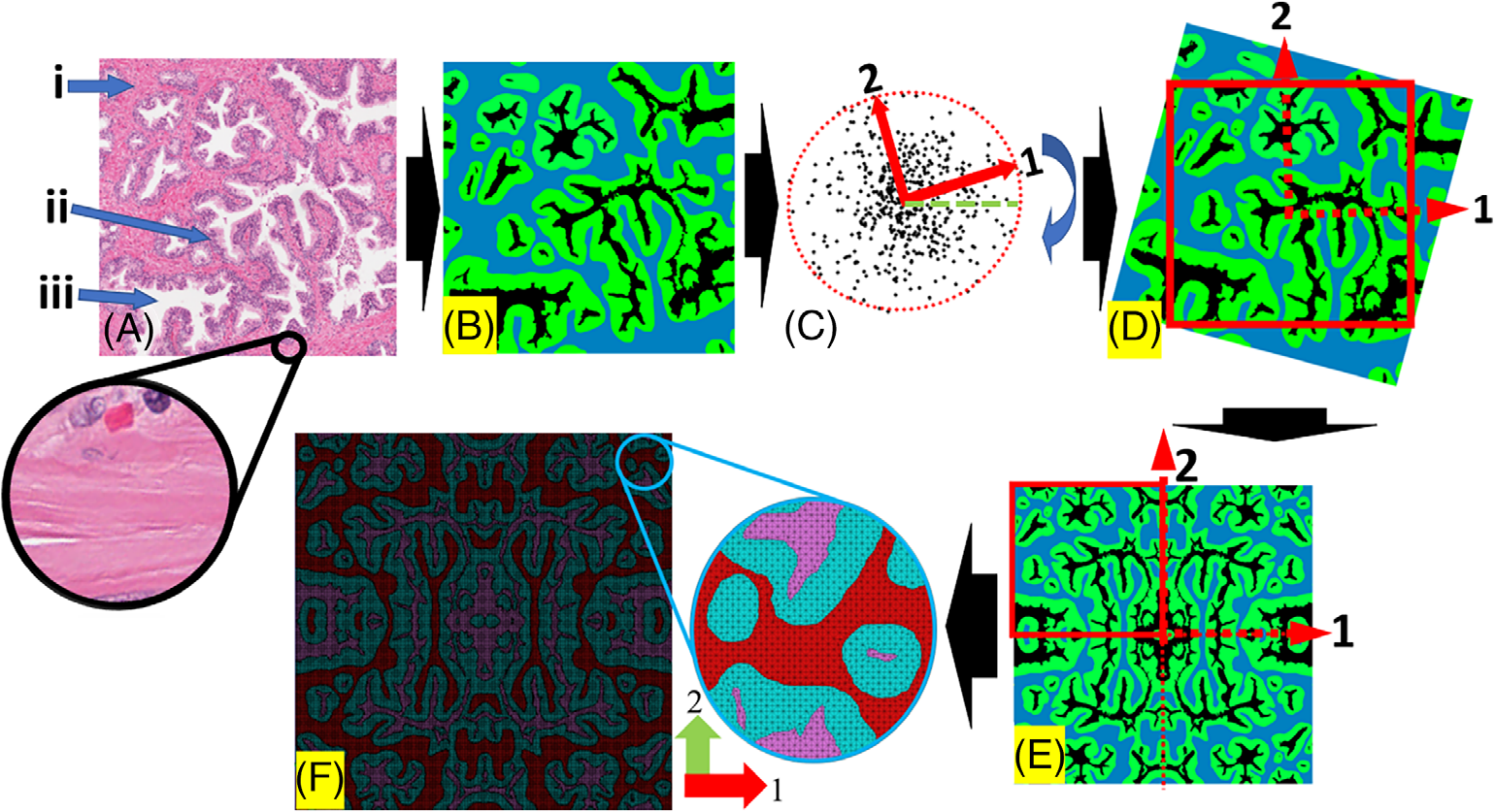
Schematic of the modelling process. (A) Raw histology image—the region of interest (RoI); (B) segmented image showing three tissue constituents; (C) tissue fabric tensor analysis identifying the principal directions of the microstructures; (D) rotating the RoI according to the principal directions and crop into new ROI (red square); (E) mirroring to achieve the perfectly orthotropic RVE; (F) finite element mesh. Arrows in (A) indicate (i) stomal (light pink region between glands); (ii) epithelial cell compartments (dark purple area surrounding prostate acini); (iii) prostate acini (white) and circle in (A), containing multiple sub‐regions, shows little dispersion of collagen fibre orientations.

Each RVE model was then processed in QuPath,^21^ an open source code for histology image analysis. Three major constituents of the prostatic tissue, namely stromal tissue, epithelial cell compartments, consisting of basal and glandular epithelial cells, and prostatic acini were segmented. Greyscale thresholding was used for stromal tissue and prostatic acini, verified by the uropathologist. Manual segmentation and correction for epithelial cell compartments were introduced and, again, verified by the pathologist. The images were then rotated into its principal directions based on the fabric tensor analysis (detailed later). A process that helps achieve a perfectly orthotropic model whilst ensuring periodicity^22^ was followed to make the model suitable for computational homogenization formulation with periodic boundary conditions. Figure 1 illustrates the steps followed in this study to obtain the finite element model for the computational homogenization using the histology images. First, to obtain the local orientation of the collagen fibres in stromal tissue, the segmented images containing stromal tissue as foreground were processed in ImageJ using an open‐source plugin^23^ to yield a map of local orientations. The local fibre orientations were then used to account for the anisotropic hyperelastic model in stroma (detailed later). Second, for the finite element (FE) analysis, the segmented images were then imported in ScanIP (Simpleware, Synopsys), where the FE mesh was created using a mix of 2D triangular and rectangular elements. Following the mesh convergence where the number of mesh elements was increased until the apparent properties converged, the mesh density was optimized so that the geometric interfaces between the three modelled constituents were preserved, leading to a total of approximately 300–400k elements for each model. The FE model was then imported into ABAQUS (Simulia, Dassault Systemes), where all stromal elements were modelled using anisotropic hyperelasticity, each assigned with a local direction of collagen fibres using the aforementioned orientation map.

### 2.2 Material modelling of tissue constituents

Although apparent elastic properties of prostate tissue have been characterized before,^16^, ^24^, ^25^ the mechanical properties of three structural constituents have not been reported. The approach, therefore, was to adopt the data from literature that reported mechanical properties of the same tissue constituents in closely relevant tissue types.

First, the stromal tissue, due to its high content of collagen fibres, was modelled as a fibre‐reinforced hyperelastic material using the HGO (Holzapfel‐Gasser‐Ogden) model.^26^ More specifically the ABAQUS implementation of the HGO model was used. The HGO model was selected due to its ability to explicitly model the mechanical behaviour of the collagen fibres in stroma tissue. This includes explicitly modelling the orientation of the collagen fibres which were calculated in the orientation analysis and mechanical properties of the collagen fibres. In addition, the ABAQUS implementation of the HGO model has the ability to ‘deactivate’ the collagen fibre stiffness when the hyperelastic matrix is under compression due to the definition of $\left\langle E\right\rangle$ found in Equation (1). Its strain energy potential can be expressed, with neo‐Hookean hyper‐elasticity and a single group of collagen fibres, as

(1) $$\Psi_{\mathrm{HGO}}=\Psi_{N-H}+\frac{k_{1}}{2k_{2}}\left(\exp\left[k_{2}{\left\langle E\right\rangle }^{2}\right]-1\right),$$

(2) $$E=\kappa \left({\bar{I}}_{1}-3\right)+\left(1-3\kappa \right)\cdot \left({\bar{I}}_{4}-1\right),$$

where, *k*_1_ denotes the stiffness of the fibre group and *k*_2_, a dimensionless parameter, represents the nonlinear behaviour of the fibres. The quantity $\left\langle E\right\rangle$ characterizes the strain along the direction of the mean orientation of the fibre group, and is ‘deactivated’ when the hyperelastic matrix is under compression along the fibre direction. ${\bar{I}}_{1}$ is the first invariant of the modified right Cauchy‐green tensor and ${\bar{I}}_{4}$ represents the squared stretch along the fibre group direction. *κ* describes the dispersion of the collagen fibres group, which is perfectly aligned with no dispersion when *κ* = 0 and isotropic when *κ* = 1/3. Orientation analysis in the stromal tissue was conducted by dividing it into small sub‐regions, which was typically much smaller than the characteristic length scale of the stromal tissue. Each sub‐region was analysed to give the orientation of the collagen fibres in it. Based on the orientation analysis of collagen fibres in those sub‐regions and close examination of the histology images, the dispersion in all the sub‐regions was deemed to be insignificant therefore the collagen fibres in prostate stroma was modelled as a single group with no dispersion. Critically, the dispersion of the collagen fibre in stromal tissue at the larger length scale was then inherently modelled by applying different local orientations in each sub‐region, hence *κ* = 0. The mechanical behaviours of stromal tissue has been most extensively studied in cornea tissue, which is rich in stromal tissue.^27^ For the epithelial cell compartment, a neo‐Hookean hyper‐elastic model was employed here, similar to Equation (1) but without the fibre‐term:

(3) $$\Psi_{N-H}=C_{10}\left({\bar{I}}_{1}-3\right)+\frac{1}{D_{1}}{\left(J-1\right)}^{2},$$

where, $C_{10}$ is the material constant, *D*_1_ the material incompressibility parameter and *J* the elastic volume ratio. In this study, near‐incompressibility was assumed for all tissue constituents. Finally, the acinar lumen was also modelled as a neo‐Hookean hyper‐elastic material. All models were solved using finite element under plane stress conditions.

Table 1 summarizes the varying ranges of these material parameters reported in literature and such ranges were used for a comprehensive parametric study carried out for all key material parameters. These include *C*_10_ and *k*_1_ for the neo‐Hookean term and fibre term of stromal tissue, respectively, and neo‐Hookean parameters of *C*_10_ for epithelial compartment and acinar lumen. To reduce the number of possible combinations of material constants used in the parametric study, the neo‐Hookean model was used. It was also found to adequately represent the non‐linear response of the different constitutes at the strain range tested in this study.

**TABLE 1:** Material properties used in this study and their sources.

Name	Material parameter	Value and reference
Normal prostatic tissue (apparent)	Elastic modulus (compressive)	20 kPa ± 5 kPa, adopted from References 13, 16, ^a^
Stromal tissue	*C*_10_ (neo‐Hookean)	5–30 kPa, adopted from References 27, 28
	*k*_1_	10–300 kPa, fitted using data from References 7, 29, 30, 31
	*k*_2_	200, fitted using the same data as above
Epithelial cell compartment	Young's modulus (fitted with nearly‐incompressible neo‐Hookean hyperelastic model)	1–40 kPa (Young's modulus), adopted from References 32, 33, 34; equivalent to 0.192–4.801 kPa (neo‐Hookean *C*_10_) considering near‐incompressibility
Acinar lumen	*C*_10_ (neo‐Hookean)	1.92–192 kPa

^a^ A higher range of elastic modulus of ~50 kPa was reported by Reference 10 on indentation results from small tissue samples, but not used in this study.

### 2.3 Computational homogenization with periodic boundary conditions

**FIGURE 2:**
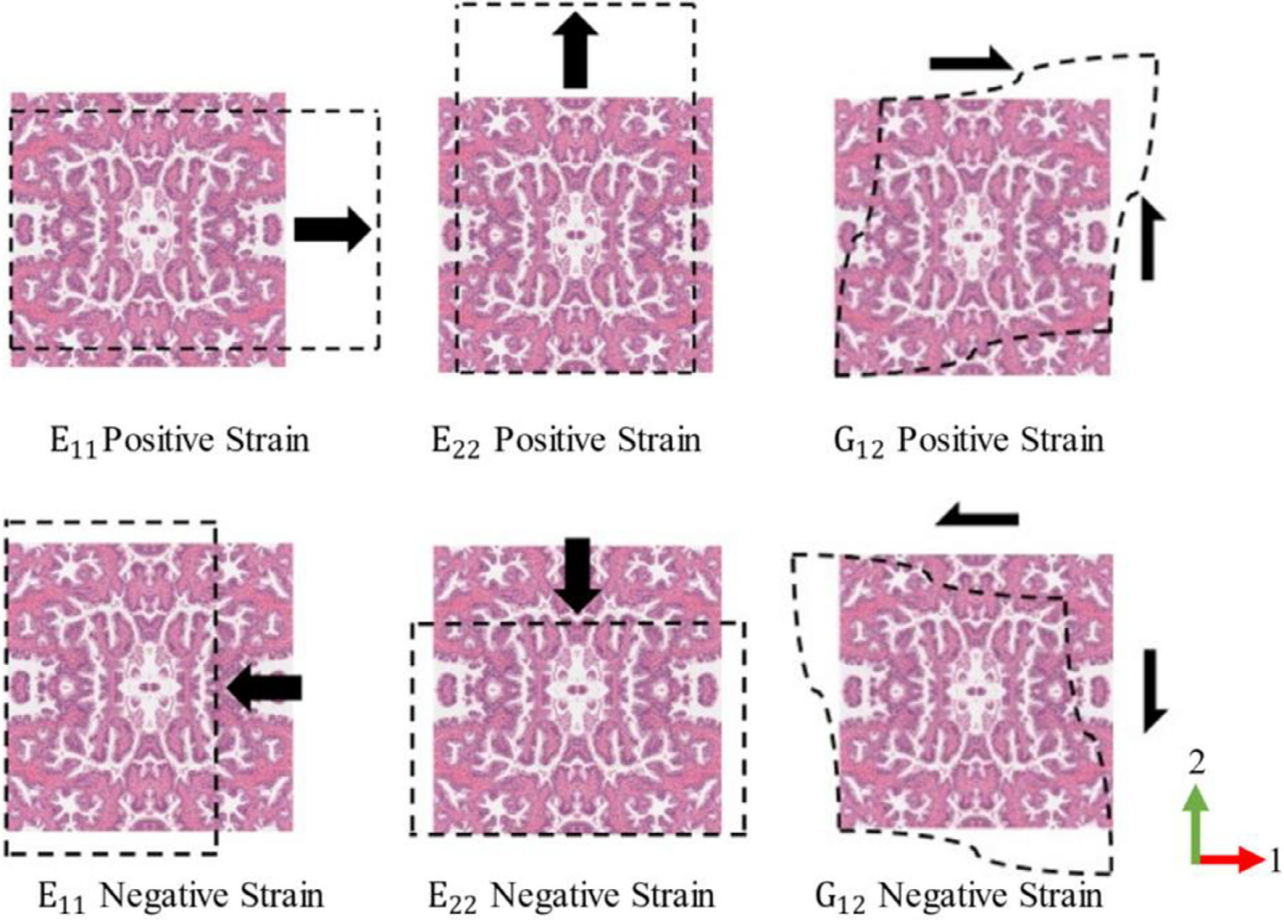
Schematic diagram of various loading conditions for computational homogenization.

To assess the apparent mechanical properties of all selected RVEs, a computational homogenization scheme with periodic boundary condition (PBC)^35^, ^36^, ^37^ was implemented. The PBC was achieved by constraining the degrees of freedom of nodes on opposing faces/edges and linking them to a reference point. These reference points were then used to apply the desired macroscopic strain to the model. For more information on how the PBC were implemented, the readers can refer to References 35, 36, 37. Three tests were conducted, including two uniaxial tests along the orthonormal directions and one shear test, as illustrated in Figure 2. A global test strain of 5% was selected to be used in this study. Each test included two variations, each with a positive or negative global test strain. As a result, two sets of apparent tissue properties for each RVE model were obtained, including the moduli *E*_11_, *E*_22_, and *G*_12_, under positive and negative global test strains, respectively.

### 2.4 Fabric tensor analysis

Fabric tensor analysis, widely used for characterizing heterogeneous materials,^38^ was employed to quantify the morphological orientation and anisotropy in the prostatic tissue. Based on the segmented images for all RVEs, three individual images were created, one for each tissue constituent (white for the specified constituent as foreground, black for the rest as background). BoneJ,^39^ an ImageJ plugin, was used to quantify the directionality of the tissue microstructure and derive its structural tensor. The method of mean intercept length (MIL) was used,^40^ where parallel lines along a random sampling direction were drawn over the foreground and the average intercept length was calculated. All MILs were illustrated by a point cloud, each point representing a MIL vector, which then represented the structural anisotropy of the chosen foreground. To quantify such structural anisotropy, the 2D point cloud was fitted by an ellipse in MATLAB, leading to two radii (*a* ≤ *b*) along the directions of the eigenvectors, and the degree of anisotropy (DA) can be written as

(4) $$\mathrm{DA}=1-a^{2}/b^{2},$$

where, DA = 0 indicates structural isotropy and DA = 1 full anisotropy. The RoIs were then rotated into its principal direction, and then cropped into squares and the fabric tensor analysis was conducted again on the final RoIs to confirm that the principal direction had not been affected by the cropping, with an allowance of maximum 2°.

## 3 RESULTS

### 3.1 RVE size

**FIGURE 3:**
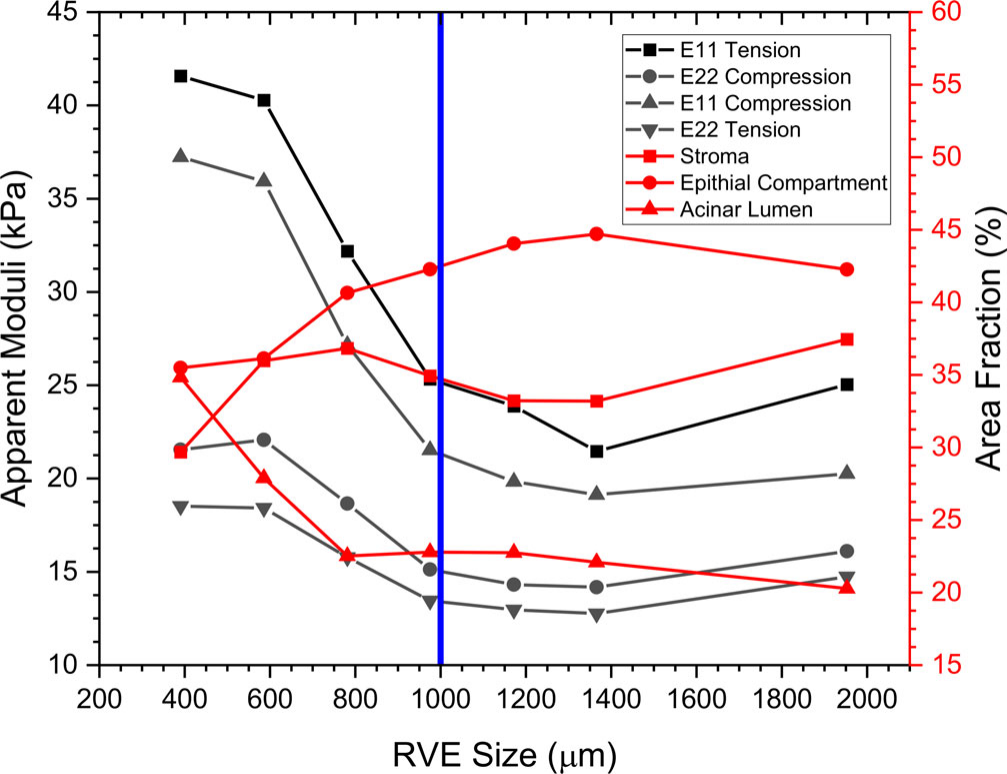
Determination of RVE size, taking into account both the apparent moduli and area fractions.

Prior to the structural and mechanical analysis, the size of an RVE was determined. This was achieved by gradually increasing the model size until the apparent properties converged. Figure 3 shows the convergence of apparent moduli, for both directions (1 and 2) under compressive and tensile loading conditions, and the area fraction of tissue constituents for model 7, chosen as a representative example, when the model size was increased from 390.6 to 1953 μm. The convergence of the area fractions was reached at a model size of approximately 1000 μm. All models were found to converge beyond the size of 1000 μm, which was then used as the desired RVE size for the subsequent sections.

### 3.2 Effect of stromal tissue

The influence of stromal material parameters on the apparent tissue properties was investigated, with a focus on the neo‐Hookean term *C*_10_, for the stromal matrix and the *k*_1_ for the anisotropic collagen fibre term. Three intermediate values were given to both *C*_10_ and *k*_1_, between the upper and lower bounds found in the literature as shown in Table 1, whilst the material properties of epithelial compartment and acinar lumen were kept constant using the benchmark values. As a result, there were a total of 25 combinations for stromal *C*_10_ and *k*_1_.

**FIGURE 4:**
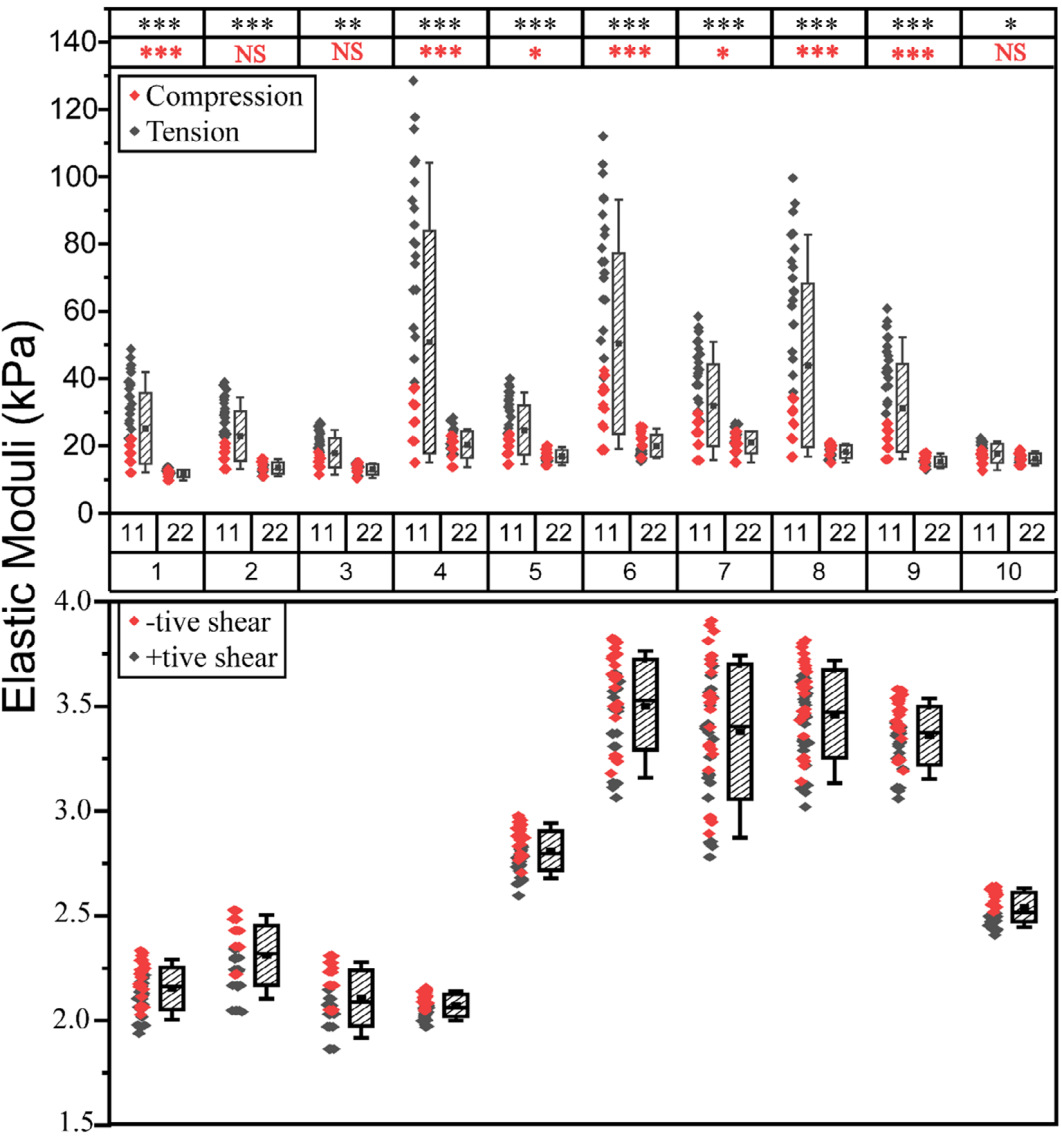
The apparent elastic and shear moduli of all chosen tissue RVEs. Differences between two directions for elastic moduli were classified as NS (not significant), * (*p* < .05), ** (*p* < .01) and *** (*p* < .001), following a standard statistical *T*‐test. The statistical significance values were colour‐coded to display the comparison between directions 1 versus 2 under tension (black, top row) and compression (red, bottom row), respectively.

Figure 4 summarizes the elastic and shear moduli of all RVEs, where results are organized with respect to the loading directions (e.g., 11 denotes the direction 1 and 22 direction 2). First, for the apparent elastic moduli, direction 1 was more prevailing. Interestingly, along the dominant direction 1, the apparent elastic moduli under the tensile loading condition were consistently higher than those under compression. However, such a trend became much less significant and even reversed in some cases along the direction 2.

#### 3.2.1 Directionality and tension‐compression asymmetry

**FIGURE 5:**
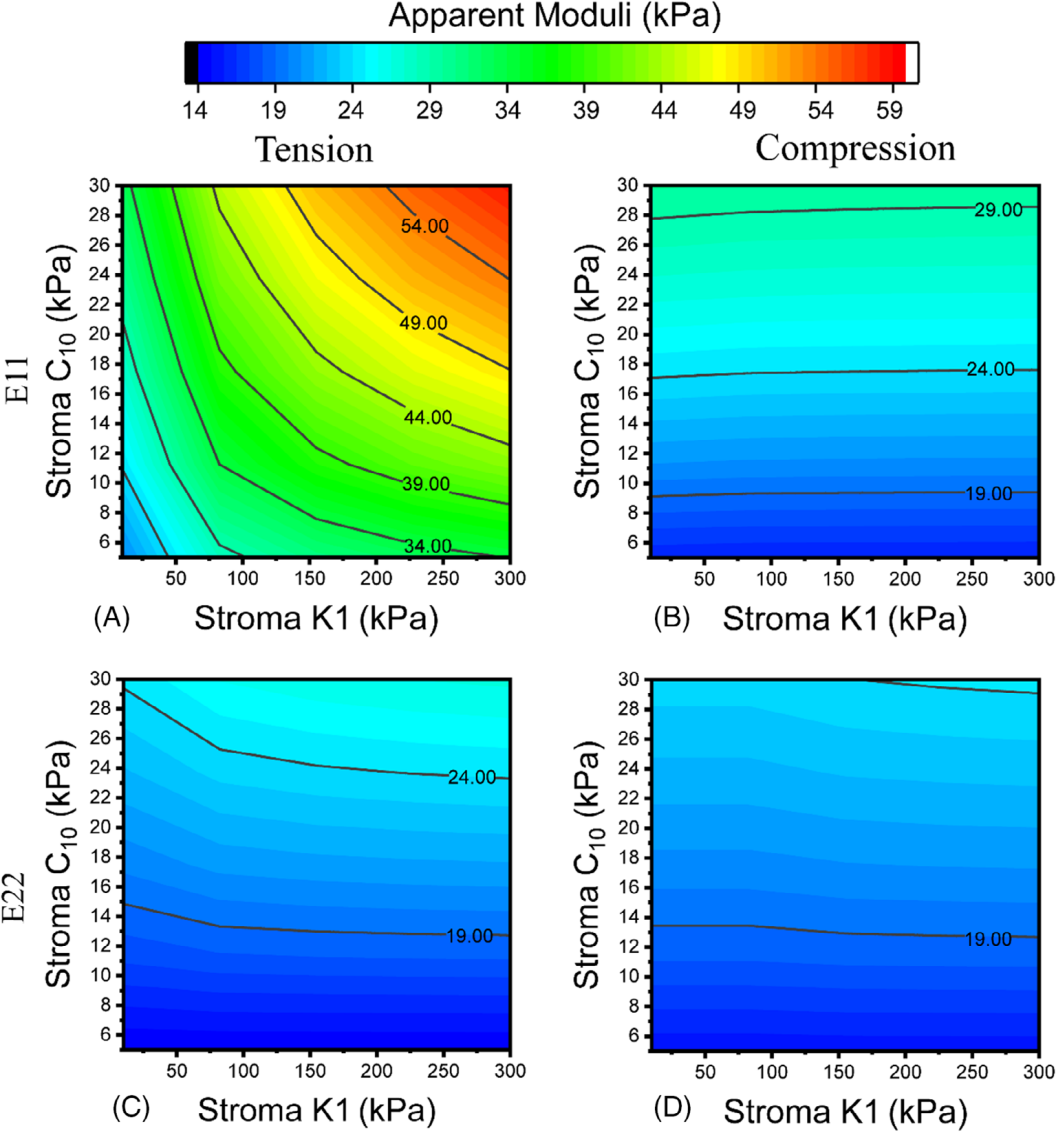
Sensitivity analysis of *C*_10_ and *k*_1_ of stroma, using RVE 7 as an example.

Figure 5 illustrates the effects of stromal tissue in the apparent elastic moduli of RVE 7 as an example. The same trend was observed consistently across all models. Similar to the results shown in Figure 4, the apparent elastic modulus displayed certain directionality, where the results along the direction 1 (*E*_11_) were greater than those along the direction 2 (*E*_22_) under tensile loading conditions. However, the difference became less significant under compression. In addition, it was evident that the apparent moduli were significantly higher when the tissue is under tension in comparison to compression.

In all cases, *C*_10_ parameter of stroma exhibited more significant impact on the apparent elastic moduli than the *k*_1_ parameter. It was clear that the effect of *k*_1_ depended on the direction of global test strain and only became prominent when subjected to tensile global test strain.

#### 3.2.2 Benchmarking with literature data

**FIGURE 6:**
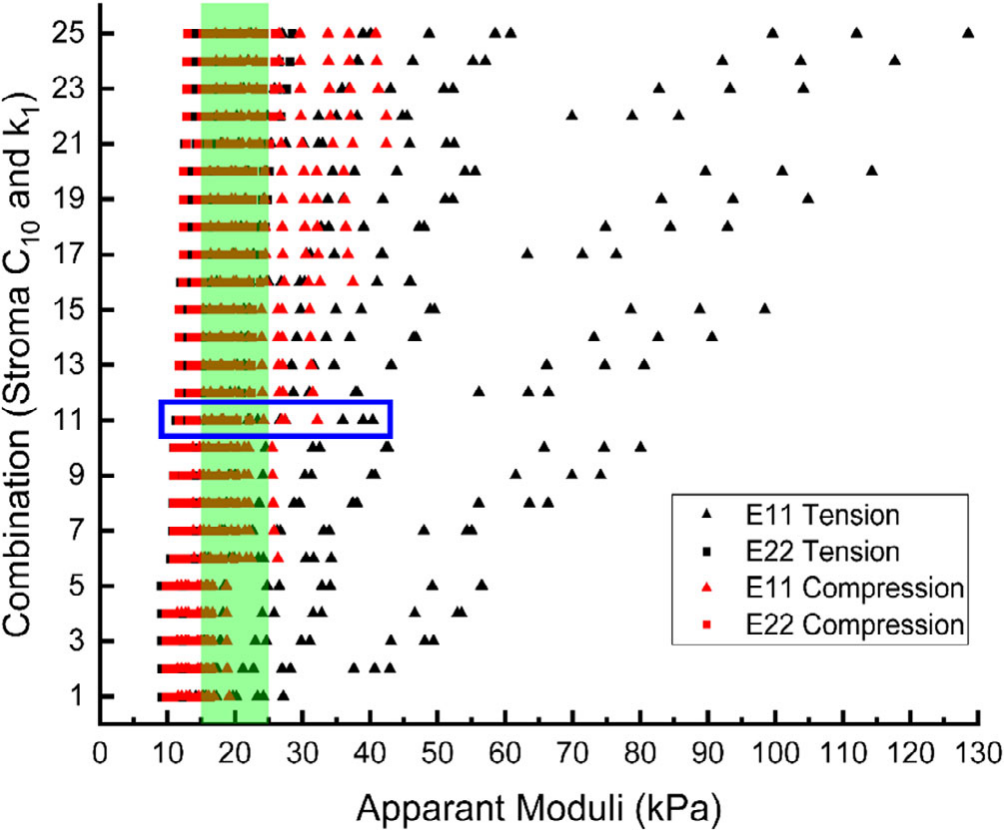
The apparent moduli of all tissue RVE models for the parametric study of stromal material parameters (*C*_10_ and *k*_1_) and the comparison against the experimental benchmark value of compressive modulus of 20 kPa ± 5 kPa (shaded in green).

As discussed above, and summarized in Table 1, an experimental range for the apparent compressive modulus of normal prostatic tissue (20 kPa ± 5 kPa) was used to benchmark the results for apparent elastic moduli. Figure 6 illustrates the apparent moduli of all tissue RVE models under both tensile and compressive loading conditions. Only the compression results were compared with the range of the compressive modulus from the literature, shaded in light green. Once again, increases in *C*_10_ and, to a less extent, *k*_1_ were found to have certain effects on the apparent moduli which, displayed greater values in tensile cases than compressive ones. Importantly, when *C*_10_ = 17.5 kPa and *k*_1_ = 155 kPa, the apparent compressive moduli were found to be most suitable in order for the apparent compressive moduli to yield a value within the range reported in the literature. As a result, those values for the stromal material parameters were chosen for the subsequent analysis in this study.

#### 3.2.3 Correlation between apparent moduli and area fractions of tissue constituents

**FIGURE 7:**
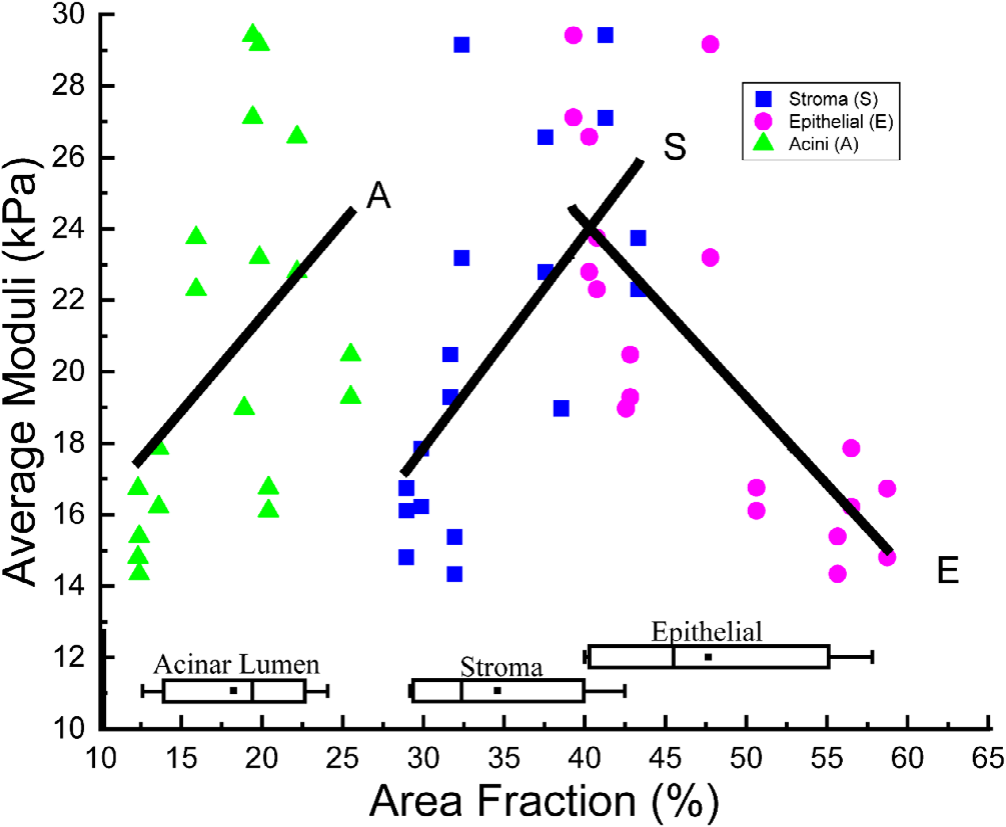
The correlation between the apparent elastic moduli, averaged between directions 1 and 2, of all prostatic tissue RVEs and the area fractions of three tissue constituents. Linear regression was obtained for each dataset. *R* ^2^ = 0.41 (S), 0.54 (E) and 0.22 (A).

The area fractions of all three tissue constituents were extracted from the histology models and compared with the apparent elastic moduli, as shown in Figure 7. Epithelial compartment was found to have the highest area fraction, followed by stroma, and finally the acinar lumen. Positive correlation for the stromal tissue (*R* ^2^ = 0.41) and negative correlation for the epithelial compartment (*R* ^2^ = 0.54) were found, showing the contrasting effects of these two tissue constituents. The acinar lumen, on the other hand, was shown to have weaker correlation with the apparent tissue modulus (*R* ^2^ = 0.22).

### 3.3 Parametric study of epithelial compartment and acinar lumen

**FIGURE 8:**
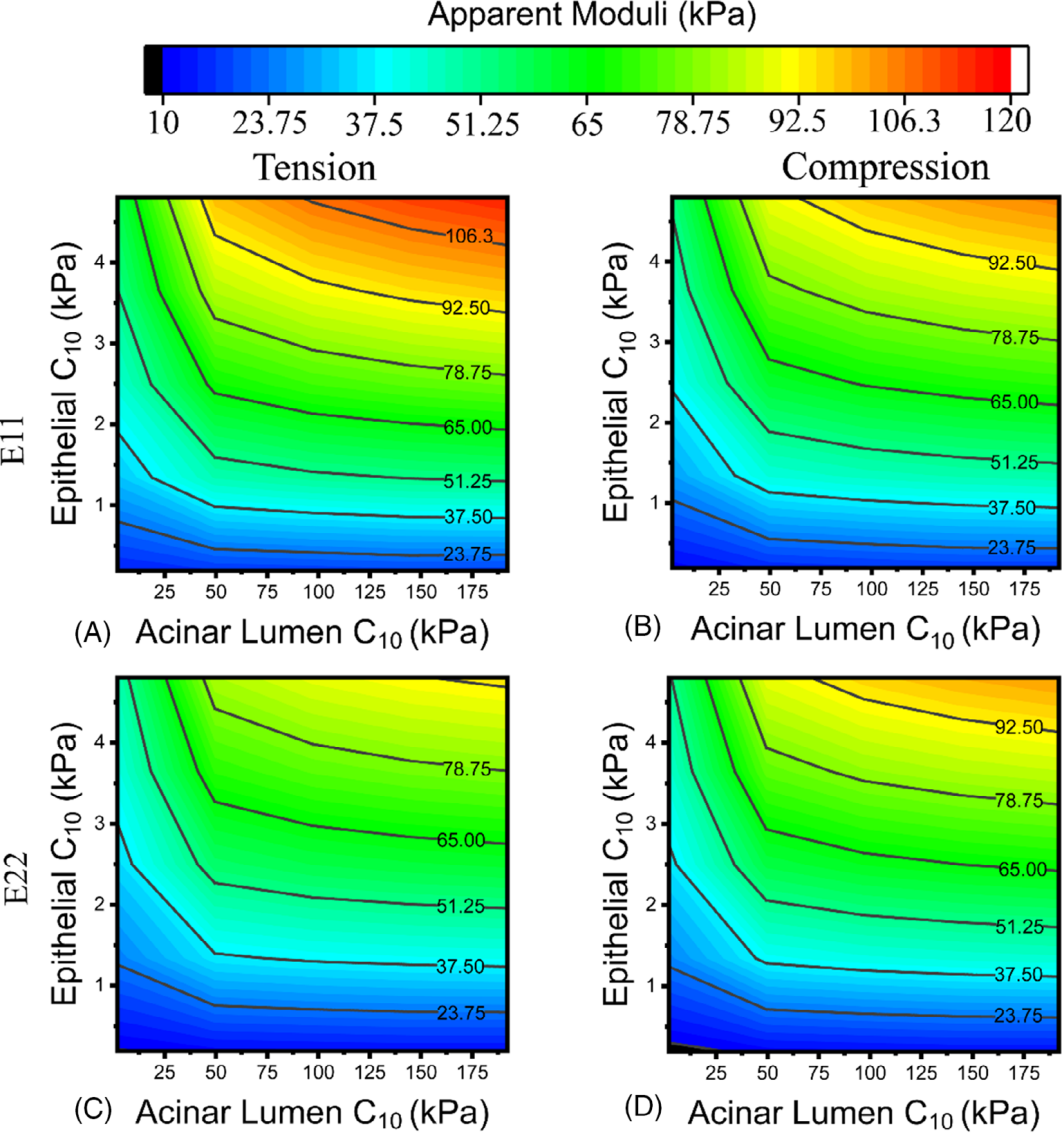
The sensitivity analysis of epithelial compartment and acinar lumen on the apparent properties of prostatic tissue, using RVE 7 as an example.

Using the benchmark values of stromal parameters *C*_10_ (17.5 kPa) and *k*_1_ (155 kPa) determined above, the effects of the epithelial compartment and the acinar lumen on the apparent tissue properties were investigated. Figure 8 illustrates their influences in the apparent tissue modulus, again using RVE 7 as a representative example. The epithelial compartment was found to have a more significant effect on the apparent moduli than the acinar lumen in all cases.

### 3.4 Fabric tensor analysis

**FIGURE 9:**
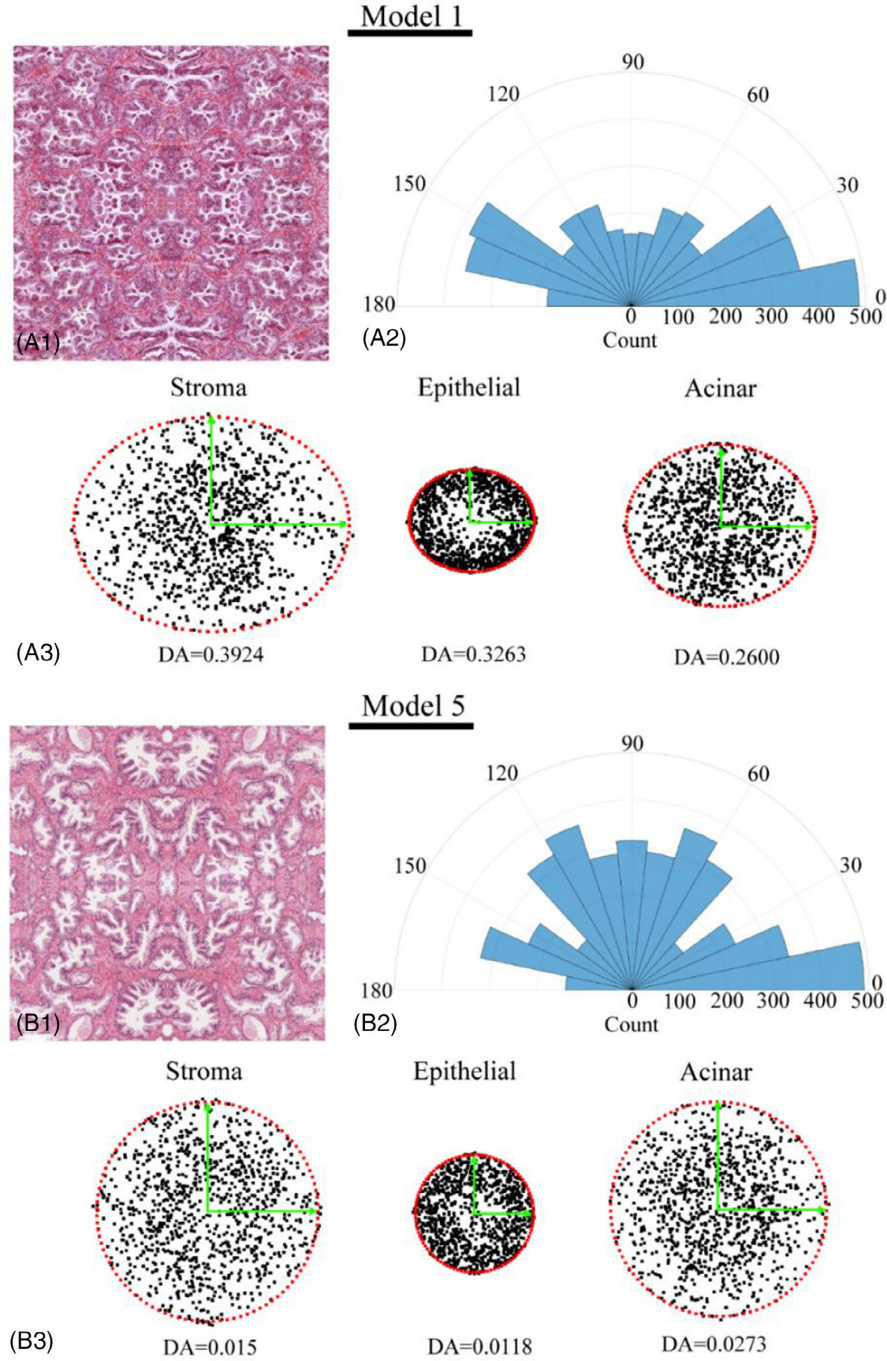
Fabric tensor analysis of two exemplar RVEs (1 and 5), including the histology images (A1 and B1), the distribution of collagen fibres in stroma (A2 and B2), and the point clouds of MILs (A3 and B3) for all three constituents with the degree of anisotropy (DA) values and major/minor axes of the fitted ellipses.

Fabric tensor analysis was carried out for individual tissue constituents in all RVEs, as shown in Figure 9. Two examples, RVEs 1 and 5, were chosen, due to them having distinct DA values, indicating significant differences in microstructural anisotropy. Point clouds of MILs with the estimated values of DA are presented for each model visually illustrating the microstructural anisotropy. Among all models, the stromal tissue has consistently higher degrees of structural anisotropy than epithelial compartment and acinar lumen. Between the two examples below, model 1 displayed significantly higher anisotropy than model 5. However, their collagen fibre orientation in stroma displayed similar distributions.

## 4 DISCUSSION

The mechanical behaviour of many soft tissue types have been studied in the literature, providing useful insights, not only into their apparent properties^41^ but also the relation to the underlying tissue microstructures.^42^ Despite the number of studies on the elastic modulus of prostate tissue measured at the ‘apparent level’, few studies have evaluated such a structure–property relationship in human prostate tissue, with a view of quantifying the contributions of tissue constituents to the complex apparent mechanical behaviours. This is key to the quantitative understanding of microstructural and mechanical heterogeneity and the role of stromal tissue, in particular the orientation of collagen fibres found in the stromal tissue, in determining the anisotropic and tension‐compression asymmetric characteristics. In our previous work,^18^ we presented the necessity of addressing this for the purpose of clinical diagnosis of prostate cancer using tissue mechanical properties as diagnostic indices. A similar need was also highlighted by,^10^ although the focus was on the material behaviour under finite deformation. Furthermore, there is growing interest in understanding the microstructural and micromechanical evolution of stromal tissue in the development of prostate cancer. Although this study, as it currently stands, did not involve cancerous tissue, its quantification of the micromechanical influence of the stromal tissue on the apparent properties of prostate tissue could be investigated with the same computational framework developed here.

Normal prostate tissue is highly heterogeneous, involving mainly three structural and mechanically‐significant constituents, namely stromal tissue, epithelial compartments and acinar lumen, each manifested in different characteristic microstructures. Stromal tissue, in particular, has a highly inter‐connected network over the entire prostate, due to one of its key functions that is to mechanically support the glandular and acinar structures.^43^ The RVE size determined in Figure 3, often incorporating at least 6–10 typical sizes of acinus, reflected on such structural heterogeneity in prostate tissue. It should be noted here that the chosen size of RVE in this study may only be applicable to normal prostate tissue and may be different for other tissue with different microstructural characteristics such as prostate cancer.

Similar to the microstructural heterogeneity that can be clearly observed from histology with the examples illustrated in Figure 9, the predicted apparent moduli of the prostate tissue also displayed significant heterogeneity and, more importantly, anisotropy. Crucially, the directions and degree of anisotropy in the apparent moduli in Figure 4 were related closely to the structural anisotropy revealed by the fabric tensor analysis. As shown in Figure 9, although the stromal tissue and acinar lumen had consistently greater averaged sizes of compartmental areas illustrated by larger point clouds of MIL, the fitted ellipses for all three constituents had similarly directionality, aligned along the directions 1 and 2. This confirmed that the RVE was indeed aligned correctly along the principal directions following the step described in Figure 1C,D. When the degree of anisotropy (DA) was significant, for example, in RVE 1, the direction of the major axis of the fitted ellipse, that is, direction 1, corresponded well to the more dominant direction of apparent moduli. In contrast, when the DA was less significant, the same can still be observed, due to the prevailing direction of the collagen fibres along the direction 1. This indicates that, in addition to the influence of the stromal microstructural orientation on the tissue anisotropy, the orientation of the collagen fibres in the stroma could also play a significant role. These results indicated a clear relationship between the microstructural anisotropy of prostate tissue, particularly the directionality of its stromal network, and its anisotropy of the apparent properties. The more prevalent the structural anisotropy is, the more significant the directionality of the elastic moduli could become. Furthermore, it is important to point out here that there is ample literature on the microscopic diffusion anisotropy in prostate tissue obtained from the diffusion‐weighted and diffusion tensor imaging (DWI/DTI).^44^, ^45^ Such diffusion anisotropy has been shown to be sensitive to the directionality of the tissue morphology.^46^ Thus, with the tissue microstructure as a ‘bridge’, the anisotropy of the tissue diffusivity may also be somewhat linked and correlated with the anisotropy of apparent elastic properties of the prostate tissue.

In Figure 4, all RVE examples had consistently higher tensile moduli than those under compression along the direction 1, however such a trend was less significant along the direction 2. This tension‐compression asymmetry is caused by, and highly correlated with, the directionality and degree of anisotropy of the stromal tissue, due to its stiffening behaviour arising from the collagen fibres. Physically speaking, the fibre‐induced reinforcement may only occur when the stromal tissue in that region is subjected to a tensile stretch along its local orientation of the collagen fibres, which can be seen in the histology images to correspond well with the orientation of the stromal ‘channels’. The collagen fibre network is also well aligned along the direction of such stromal channels. Since the more prevailing principal direction (i.e., direction 1) of the fabric tensor for stromal tissue is an estimated representation of the dominating direction of its microstructure at the ‘apparent’ scale, the direction at which the tension‐compression asymmetry was found to be most significant is indeed along the major principal direction 1, as shown in Figure 4.

The elastic moduli of normal prostate tissue have a significant degree of variability according to literature, shown in Table 1, with reported values mostly ranging from 10 to 60 kPa, heavily affected by the tissue conditions and the characterisation methods. Given that there have been no studies that characterized the prostate‐specific tissue constituents, the approach was to carry out parametric studies to quantify their roles in the apparent mechanical properties benchmarked against the range of key material parameters from relevant studies (see Table 1). The stromal tissue was analysed first, with varying material parameters of its hyperelastic matrix and the collagen fibres, as shown in Figure 5. Using the compressive elastic moduli reported in the literature, against which all the results were benchmarked, the stromal material properties were determined. It might be noted that, if benchmarked against a higher value from the literature, for example, 55 kPa ± 14 kPa,^10^ a different set of material parameter values may be chosen, but all conclusions made from the parametric study would still be valid and consistent. Using a similar approach, the effect of epithelial compartments was seen (in Figure 8) to be more significant than acinar lumen. However, it should be stressed here that the effects of all tissue constituents must not be viewed separately, due to the fact that they are competing for their area fractions. The finding of area fractions of prostate tissue constituents in Figure 7 is consistent with the values reported in a previous study.^47^ It is therefore not surprising to observe that stroma and epithelial compartments have greater effects on, and correlation with, the apparent properties, due to the fact that these two constituents have greater area fractions as confirmed by the results in Figure 7.

This study, as it currently stands, has a few limitations, thus presenting opportunities for future development. First, the mechanical properties of the prostate tissue constituents have not been directly characterized before in literature. To ensure the relevance of this study to the prostate tissue, mechanical properties from carefully‐selected studies with relevant characterization methods were adopted and the apparent tissue moduli in the parametric study were benchmarked against these. Second, to the best of the authors knowledge, no literature values for tensile modulus of prostate tissue are available. As a result, a comparison against literature for tensile results could not be conducted. Third, the homogenization framework developed in this study focused on the quasi‐static behaviours of the prostate tissue. Although this choice was, in part, due to the fact that the benchmark tissue properties were also derived from quasi‐static experiments from literature, it is expected that the future work will explore time‐dependent behaviours, for example, viscoelasticity, of the prostate tissue. Third, 2D histology images were used to reconstruct the tissue RVE models. Whilst the prostate tissue would no doubt have a certain degree of heterogeneity along the direction perpendicular to the histological sectioning plane, this study still presents novel insights into the structure–property relationship. It is possible to adopt a similar homogenization scheme as proposed in this study and apply it to 3D models reconstructed from serial sectioning, for example, tissue microarray, and it is being carried out in an on‐going study by the authors. Fourth, only the collagen fibres in the histology sectioning plane were characterized. This was a simplification as the stroma and its embedded collagen fibre have a 3D network in normal prostatic tissue – this is also being addressed in our on‐going study. Fifth, non‐linear hyperelastic material behaviours were used for the tissue constituents but only the apparent modulus was compared against literature which has no reported values on prostate hyperelasticity. This was due to the limited amount of mechanical characterisation data in literature on prostate tissue because of the difficulty of performing experimental tests at large deformation tensile/compression due to the tissue requiring sample preparation, for example, tissue cutting. Due to the nature of glandular tissue, sample preparation would change the tissue condition so drastically that, for example due to loss of interstitial liquid, would render the experimental characterisation invalid. We decided to keep our study in the low strain regime (up to 5%), therefore the material behaviours still remain within the ‘toe’ region of the hyperelastic responses with significantly less nonlinearity compared to the typical behaviours at high strain (e.g., >50%). As a result, we found that the derived apparent modulus within 5% of the strain is rather linear with respect to the strain applied. A future experimental study is planned to investigate the large strain behaviours of the prostatic tissue and the characterization data will be used for a similar computational study. Finally, whilst the present study focused on normal prostate tissue, in a future study we will use the proposed methods to examine the relationship between the apparent properties of cancerous tissue in prostate and its microstructural indices such as Gleason scores.

## 5 CONCLUDING REMARKS

This study presented a computational framework for quantifying the apparent mechanical properties of prostate tissue models reconstructed from histology images. A parametric study was carried out to quantify the roles of three major tissue constituents, namely the stromal tissue, epithelial compartment and acinar lumen in the apparent elastic modulus of prostate tissue, benchmarked with the literature data. The results presented confirmed the existence of the mechanical anisotropy and the tension‐compression asymmetry in normal prostate tissue and, importantly, revealed the critical role of the tissue microstructures in dictating such complex mechanical behaviours. Specifically, prostate tissue constituents were shown to have significant effects on the apparent tissue properties. The correlation between the stromal tissue orientation and the preferential direction of the apparent properties suggested an essential role of stromal tissue in determining the directions of anisotropy and the compression‐tension asymmetry characteristics. The proposed approach has promising potential in quantitative understanding of tissue mechanical properties with evolving microstructures, particularly subject to pathological conditions such as prostate cancer and benign prostatic hyperplasia, for the purpose of clinical diagnosis that is based on the tissue mechanical properties.^48^

## CONFLICT OF INTEREST STATEMENT

The authors declare no conflict of interest. All funding received for this study has been acknowledged.

## Data Availability

The data that support the findings of this study are available on request from the corresponding author. The data are not publicly available due to privacy or ethical restrictions.
